# Supplementary material for: Patterns of Intron Gain and Loss in Fungi
Source: PLoS Biol. 2004 Nov 30;2(12):e422. doi: 10.1371/journal.pbio.0020422 (PMC532390; doi:10.1371/journal.pbio.0020422)
Supplement: Table S1 — Also available at http://genes.mit.edu/NielsenEtAl/. (4.3 MB ZIP). [file pbio.0020422.st001.zip › NielsenEtAl/html/266.html]

AN1775.1.NCU05644.1.MG03759.1.FG02191.1


```
 CLUSTAL W (1.82) Multiple Sequence Alignments - Introns Inserted


Sequence 1: MG03759.1	636 aa
Sequence 2: FG02191.1	582 aa
Sequence 3: NCU05644.1	632 aa
Sequence 4: AN1775.1	582 aa
Alignment Length: 666 aa
Number Identitical Residues: 265 aa
Alignment Score (without introns) 14245


MG03759.1 	---------MQSLFAFLLFPLL----------------AAAQYHEQLVLRPLPLSALLAS
NCU05644.1	MRPSFPSSFLLSLLSLLCFSPYTAIANASSSPADSISNSRSDYHEQLILRPLPLSTLLAS
FG02191.1 	---------MRRFLAYLLLAITNGFALTSA--------AASGYHEQLTLRPLPLSQLLAS
AN1775.1  	-------MTIIYILALAIAALSVG-------------SAASNYHENLLLQPLPPSSLLAS
          	         :  :::    .   .             .: : ***:* *:*** * ****

MG03759.1 	FNFRANTTIADFEAHNYRLFPRSLAQILQHAGTRELHLRFGLGRWDAESWGARPWDGTRE
NCU05644.1	FNFRSNTSLSAFEAHNFRFFPRSLGQILEHAGTRELHLRFSLGRWDAESWGARPWNGARE
FG02191.1 	FNFRANTSIADFEAHNFRLFPRSLAQILQYAGTRELHLRFTLGRWDAQSWGARPWDGTRE
AN1775.1  	FNFRSNATQTSFQQQHFRYFPRALGQILQHANTRELHLRFTTGRWDSESWGSRPWDGAKE
          	****:*:: : *: :::* ***:*.***::*.********  ****::***:***:*::*

MG03759.1 	GGTGVELWAWLDASTDEE2ADRKWLTLTNALSGLFCASLNFIDGTRTTRPVMSFQPEGDH
NCU05644.1	GGTGVEMWAWLEADTDEE2ADRKWLTLTNALSGLFCASLNFIDSTRTTRPVMSFQPEGNH
FG02191.1 	GGTGVELWAWLDAETDAE~ADEKWLILTNALSGLFCASLNFIDETRTIRPVMSFQPDGHH
AN1775.1  	GGTGVELWAWIEAADDEI2AFSKWITLTQSLSGLFCASLNFIDSTRTTRPVASFEATGHH
          	******:***::*  *   *  **: **::************* *** *** **:. *.*

MG03759.1 	PNVTAANMHLLYGTLPREVVCTENLTPFLKLLPCKGKAGIASLLDGHKLFDASWQSMAID
NCU05644.1	IDANATNMHLLYGVLPHEVVCTENLTPFLKLLPCKGKAGIASLLDGHKLFDSSWQTMAID
FG02191.1 	SNSSLANTRLLHGVLPHEVVCTENLTPFLKLLPCKGKAGIATLLDGHKLFDASYQSMAID
AN1775.1  	VDS--DNLHLLHGTLPGEVVCTENLTPFLKLLPCKGKAGVASLLDGHKLFDAAWQSMAID
          	 :    * :**:*.** **********************:*:*********:::*:****

MG03759.1 	VRPVCSEDGGCVLEIEQTIDMVLDLDRAKRPRD1NPIPRPP~PSEELKCDTTKEYHNEHT
NCU05644.1	IRPVCPEDGECVLQIEQSIDMVLDIDRSKRPRD1NPIPRPP~PAHEMKCNTSKPYHAHDT
FG02191.1 	VRPKCDTDGECFLEMEETVDMVLDINRSKRPQN1NPIPRPP~PSHELLCDTSKPYHSDNT
AN1775.1  	VRPVCPSSGECLMQIDQTVDIVMDIERSKRPR-~------V1PADKLVCDTSKSYHARDT
          	:** *  .* *.:::::::*:*:*::*:***:          *:.:: *:*:* **  .*

MG03759.1 	CFPADYTAHQDWTLSKVFGRSMQGSCPLANSDVPPVCMQIPESRSVYTSEGVHEIKNPDG
NCU05644.1	CFPTDSAANQDWSLSQIFGKPIKGTCPLTDPNVSPVCIQVPDSRIVFASEGAVEKKNPDQ
FG02191.1 	CFPADSLDGQDWSLSQVFGRSIKGTCPLTDPDIPPVCVQIPQSRDIYTTEGAREIRSQDG
AN1775.1  	CYPLETTAAKSWSISELFGRTVSGSCPLMDTQEKTVCLRVPPTQEVRPSPELVNARFYDQ
          	*:* :    :.*::*::**:.:.*:*** :.:  .**:::* :: : .:    : :  * 

MG03759.1 	RSRCFKVDDGALVELVLAEPSQESSAHPESPGADFESRGLLKPEVPALYAERSLTGHGAE
NCU05644.1	TSRCFDFPAENDFEIFLPAPEAEDGS--------VVIEHVVKPETPLLYAERSFTGHGQE
FG02191.1 	NSRCYNLDANSELALMLVKPESQD-----------EDKILNKPETPLLYADRSFNGHGQE
AN1775.1  	FTRCYTPPSSEPFDLDVPEQAAIN------------ITSSVPLEEPVLHAERTIVGHGQE
          	 :**:       . : :      .                   * * *:*:*:: *** *

MG03759.1 	RGGVQTIFTNPSPDKEIEFVYMESLPWFMRIYLHTLTARVADSEIDSRGSTVSAGQAQAK
NCU05644.1	RGGVQTILRNPSPDQEVEFIYMESLPWFMRIYLHTISARVDGQPGKA-------------
FG02191.1 	HGGVQAILSNPS-DTDVEFVYMESLPWFMRIYLHTLSARIADAPLSN-------------
AN1775.1  	RGGMRIIFDNPSSEKPVDFIYFESLPWFLRPYIHTLQSTITGPDGVRRQAP---------
          	:**:: *: ***.:  ::*:*:******:* *:**: : : .       :.         

MG03759.1 	GDKKVIQEIYYRPALDRARGSQVEVRMRVPPASTVFLTYDFEKSILRYTEYPPDANRGFD
NCU05644.1	--DSLIQEVYYRPAVDRARGTQLEVRMTVPPASTVFLTYDFEKSILRYTEYPPDANRGFD
FG02191.1 	-SSDLIKEIYYRPALDRARGTQLELRMRIPPHCTVFLTYDFEKSILRYTEYPPDANRGFD
AN1775.1  	-TTDFIRETFYRPGVDRERGTQLELALSVPPASTVTLTYDFEKAILRYTEYPPDANRGFN
          	   ..*:* :***.:** **:*:*: : :** .** *******:***************:

MG03759.1 	VASAVVTILNSNQSS-----STSSRNANLRTASVLLSLPTPDFSMPYNVIILSSTAIALG
NCU05644.1	VAAAVITILNRDNKSGGNGRNSRFAAYNLRTTTLLCNLPTPDFSMPYNVIIFTSTAIALA
FG02191.1 	IAAAVITTLEP-------------KVLNIRTTTLLLYLPTPDFSMPYNVIIFTSTAIALA
AN1775.1  	VAPAVIKIAAS-------------KPIYIRTTSLLLPLPTPDFSMPYNVIILTSTVIALA
          	:*.**:.                     :**:::*  **************::**.***.

MG03759.1 	FGGLFNILVRRFVGADEGPRPGLANGLARLKQKIKGKFGANKTPGKDDAPTLASAGDQGA
NCU05644.1	FGGMFNILVRRFVAANEGPESGLKKGVNKLKAKVAGFLKKGK---KESAPAVAAEEEKKM
FG02191.1 	FGGLYNILVRRFVGADEAQGMALKAKIIGLIARLKGKVGKQ-------------------
AN1775.1  	FGSIFNLLVRRFVTVEEAAALRAQT----FKGRLGGKIVALRDRIRGKSAKVE-------
          	**.::*:****** .:*.           :  :: * .         .:.          

MG03759.1 	DGSKQQALT
NCU05644.1	E--------
FG02191.1 	---------
AN1775.1  	---------
          	
```
